# Contributions of Interleukin-1 Receptor Signaling in Traumatic Brain Injury

**DOI:** 10.3389/fnbeh.2019.00287

**Published:** 2020-01-21

**Authors:** Jason G. Thome, Evan L. Reeder, Sean M. Collins, Poornima Gopalan, Matthew J. Robson

**Affiliations:** ^1^Department of Anesthesia and Critical Care, Division of Biological Sciences, College of Medicine, University of Chicago, Chicago, IL, United States; ^2^Division of Pharmaceutical Sciences, James L. Winkle College of Pharmacy, University of Cincinnati, Cincinnati, OH, United States

**Keywords:** traumatic brain injury, interleukin-1, interleukin-1 receptor, cytokine, microglia, astrocyte

## Abstract

Traumatic brain injury (TBI) in various forms affects millions in the United States annually. There are currently no FDA-approved therapies for acute injury or the chronic comorbidities associated with TBI. Acute phases of TBI are characterized by profound neuroinflammation, a process that stimulates the generation and release of proinflammatory cytokines including interleukin-1α (IL-1α) and IL-1β. Both forms of IL-1 initiate signaling by binding with IL-1 receptor type 1 (IL-1R1), a receptor with a natural, endogenous antagonist dubbed IL-1 receptor antagonist (IL-1Ra). The recombinant form of IL-1Ra has gained FDA approval for inflammatory conditions such as rheumatoid arthritis, prompting interest in repurposing these pharmacotherapies for other inflammatory diseases/injury states including TBI. This review summarizes the currently available preclinical and clinical literature regarding the therapeutic potential of inhibiting IL-1-mediated signaling in the context of TBI. Additionally, we propose specific research areas that would provide a greater understanding of the role of IL-1 signaling in TBI and how these data may be beneficial for the development of IL-1-targeted therapies, ushering in the first FDA-approved pharmacotherapy for acute TBI.

## Introduction

Traumatic brain injury (TBI) is a significant clinical health problem. TBI is currently a leading cause of death and disability within the United States (US) (Centers for Disease Control and Prevention, 2014) translating to an annual economic cost of $76.5 billion (Centers for Disease Control and Prevention, 2019). Compounding this problem is the lack of FDA-approved treatments for acute injury, consequentially driving long-term costs due to the chronic effects of TBI. These chronic effects can include epileptogenesis, cognitive deficits, and neuropsychiatric conditions (McIntosh et al., 1996; Bombardier et al., 2010; Hart et al., 2011, 2016).

Depression is the most frequent neuropsychiatric disorder post-TBI, its incidence unrelated to degree of injury severity (Fann et al., 2013). Depression is linked to the high suicide rates following TBI, three to four times that of the general population (Fleminger, 2008; Alway et al., 2016; Ouellet et al., 2018). Cognitive deficits are inherent in TBI, including deficits in attention, working memory, and processing speed, among other issues (Cicerone, 1996; McAllister et al., 1999; McAllister et al., 2001; Chan, 2002). Post-traumatic epilepsy (PTE) is common following TBI (Fleminger, 2008), with an incidence rate between 10 and 35%, an effect positively correlated with severity of injury, and negatively correlated with age at insult (Appleton and Demellweek, 2002; Frey, 2003; Ates et al., 2006; Statler et al., 2009; Arndt et al., 2013). Management of PTE has proven difficult due to a lack of therapies for prevention/occurrence of PTE following neurotrauma (Barlow et al., 2000). It is possible that targeting acute molecular processes of TBI may prove an effective strategy for minimizing the emergence of depression, cognitive decline, and PTE post-TBI, thereby circumventing the lack of currently available treatments for these conditions.

**FIGURE 1:**
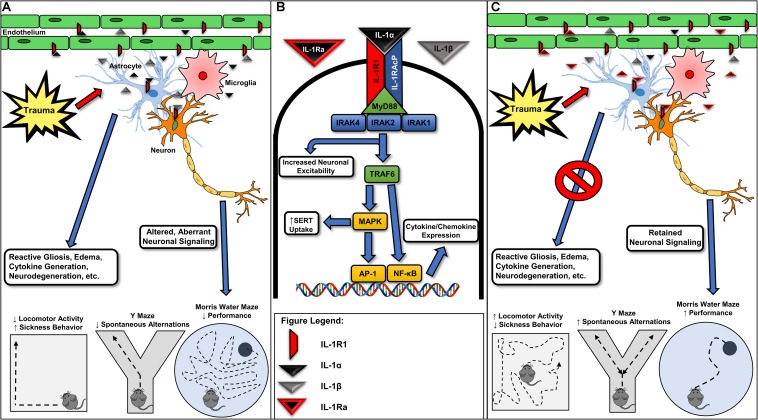
Various forms of neurotrauma induce interleukin-1 receptor-mediated signaling in the CNS. **(A)** Interleukin-1 receptor (IL-1R1) activation occurring from IL-1α or IL-1β binding in various glial cells, endothelial cells, and neuronal populations is associated with reactive gliosis, the further generation and release of inflammatory cytokines/chemokines, neurodegeneration, and ultimately, alterations in behavior. **(B)** Acute molecular signaling associated with IL-1R1 activation in various cell types of the CNS. Binding of IL-1α and/or IL-1β to IL-1R1 acts to increase gene transcription of several inflammatory mediators including IL-6 and a feed-forward loop increasing the expression of IL-1β through an activation of NF-κB and AP-1. Several proteins, including IL-1 receptor associated kinase-1 (IRAK1) and IL-1 receptor associated kinase 4 (IRAK4), are required for IL-1R1-mediated signaling and may provide further drug targets for pharmacotherapy development. IL-1-mediated activation of IL-1R1 is known to rapidly increase neuronal excitability and serotonin transporter (SERT)-mediated serotonin clearance, effects contributing to seizure susceptibility and despair-like behaviors, respectively. **(C)** Recombinant IL-1Ra (Anakinra) administration has been shown in preclinical models for neurotrauma to mitigate trauma-elicited astrogliosis, edema, and cytokine generation. Further, blockade of trauma-associated IL-1R1-mediated signaling provides cognitive-sparing effects in the Y Maze and Morris water maze behavioral assays. Given promising preclinical data and the previous FDA approval of recombinant IL-1Ra, targeting IL-1R1-mediated signaling may represent a viable avenue for the pursuit of the first FDA-approved pharmacotherapy for acute TBI. IRAK2, interleukin-1 receptor associated kinase-1; IL-1RAcP, interleukin-1 receptor accessory protein; TRAF6, tumor necrosis factor receptor associated factor 6; MAPK, mitogen-activated protein kinase; AP-1, activator protein-1; NF-κB, nuclear factor kappa-light-chain-enhancer of activated B cells; MyD88, myeloid differentiation primary response protein.

The acute phases of TBI are characterized by profound neuroinflammation, increased glial cell reactivity, cytokine generation/release, and neural degeneration. Inflammatory cytokines, including interleukin-1α (IL-1α) and IL-1β, are the primary orchestrators of neuroinflammatory cascades, and many utilize receptors in a cell heterologous and/or autonomous fashion. The IL-1 receptor type 1 (IL-1R1) is the primary functional target of three endogenous ligands, IL-1α, IL-1β, and IL-1 receptor antagonist (IL-1Ra) (Symons et al., 1995). Upon activation, IL1-R1 recruits IL-1 receptor accessory chain protein (IL-1RAcP), also known as IL-1R3, forming a heterotrimeric complex with IL-1R1 and the bound ligand (Casadio et al., 2001). Toll/IL-1 receptor (TIR) domains within the cytoplasm form a complex with the myeloid differentiation primary response gene 88 (MYD88) adapter protein, subsequently recruiting IL-1 receptor-associated kinase 4 (IRAK4) (Burns et al., 1998; Xu et al., 2000; De Nardo et al., 2018). Once recruited, IRAK4 trans-autophosphorylates, recruiting and phosphorylating IRAK1, and forming a complex with tumor necrosis factor receptor-associated factor 6 (TRAF6) (Ye et al., 2002; Burns et al., 2003; Ferrao et al., 2014; Vollmer et al., 2017). The IRAK1/TRAF6 complex initiates phosphorylation of membrane-bound transforming growth factor beta-activated kinase 1 (TAK-1), leading to downstream activation of the transcription factors nuclear factor-kappa B (NF-κB) and activator protein 1 (AP-1) (Lee et al., 2002; Shim et al., 2005; Figure 1). Together, these transcription factors promote the production of a myriad of proinflammatory cytokines involved in neuroinflammation, including IL-6 and IL-1β (Collart et al., 1990; Sung et al., 1992; Serkkola and Hurme, 1993; Jeon et al., 2000; Roman et al., 2000).

The regulatory role of IL-1R1 in innate immune system activation renders it an ideal candidate to block downstream effects of the IL-1 pathway. Beneficial effects in inflammatory diseases, such as rheumatoid arthritis, have led to FDA approval of the recombinant form of IL-1Ra, Anakinra. The availability of this treatment has fueled interest in IL-1R1-mediated signaling for various disease states/disorders with an inflammatory component. IL-1Ra administration in animal models of cerebral ischemia, for example, has been demonstrated to provide neuroprotective effects (Banwell et al., 2009; Becker et al., 2014; Girard et al., 2014; Clausen et al., 2016; Pradillo et al., 2017), with Anakinra advancing to clinical trials for this indication (Emsley et al., 2005; Galea et al., 2018; Smith et al., 2018). Further, recent studies have aimed to delineate the role of IL-1R1 signaling within the central nervous system (CNS) following neurotrauma using pharmacologic and genetic means. Recent studies, emerging clinical trials, and potential future directions specific to IL-1R1 signaling in the context of TBI are the primary focus of this review.

## IL-1R1 Localization in the CNS

While expression of IL-1R1 is ubiquitous throughout the brain, there are regions of dense expression that provide insight to abnormal behaviors believed to be associated with IL-1R1-mediated signaling. Autoradiography labeling of IL-1α and IL-1β in the mouse brain resulted in concentrated labeling of the granule cell layer of the hippocampus, choroid plexus, and pituitary gland (Takao et al., 1990; Ban et al., 1991). These studies have been corroborated via regional *in situ* hybridization of IL-1R1 mRNA within the dentate region of the hippocampus, choroid plexus, and pituitary gland, as well as in the raphe nuclei (Cunningham et al., 1991, 1992).

Functional IL-1R1 expression has been demonstrated in astrocytes, microglia, neurons, and endothelial cells (Liu et al., 2019). IL-1R1 mRNA is expressed at high levels in cultured astrocytes, and radiolabeled IL-1α has been demonstrated to bind to primary astrocyte cultures (Ban et al., 1993; Tomozawa et al., 1995; Pinteaux et al., 2002), implying expression of functional IL-1R1. Further evidence of functional IL-1R1 expression in astrocytes is the release of proinflammatory factors such as IL-6 post-stimulation (Gottschall et al., 1994). IL-1R1 expression is present within microglial populations; however, expression levels are substantially lower compared to astrocytes (Pinteaux et al., 2002). IL-1R1 appears to be expressed in microglial populations *in vivo*, as confocal microscopy analyses have demonstrated colocalization of IL-1R1 with OX-42-labeled microglia in the spinal cord (Wang et al., 2006). Binding sites for IL-1α, presumed to be IL-1R1, have also been localized to hippocampal neurons (Takao et al., 1990; Ban et al., 1991). Finally, IL-1R1 is localized to endothelial cells within the brain with activation leading to hyperthermia and cytokine signaling (Cao et al., 2001; Liu et al., 2015). The diverse localization of IL-1R1 expression within the CNS suggests a myriad of roles in the regulation of the innate immune system following neurotrauma.

## Preclinical Targeting of IL-1R1

### IL-1Ra

Much of the preclinical work aimed at delineating the roles of IL-1R1-mediated signaling in neurotrauma has utilized recombinant human IL-1Ra. IL-1Ra competitively binds to IL-1R1, thereby impeding the ability of IL-1β and IL-1α to bind to and signal through the receptor. These pharmacologic studies are significant due to their potential to repurpose an already FDA-approved therapy (Anakinra or Kineret). In a rat fluid percussion injury (FPI) model, IL-1Ra administration was shown to rescue cognitive impairment as assessed in the Morris water maze (MWM) (Sanderson et al., 1999). Acute IL-1Ra administration reduces pentylenetetrazol (PTZ)-evoked seizure hypersensitivity in a pediatric model for TBI, providing evidence that IL-1R1 blockade may be a promising avenue for preventing PTE (Semple et al., 2017; Webster et al., 2017). Similarly, the effects of IL-1Ra overexpression on the effects of TBI have been explored. Transgenic mice overexpressing IL-1Ra demonstrated significantly higher neurological recovery; evaluated by neurological severity score (NSS), a task which assesses performance on motor and behavioral tasks, in a closed head injury (CHI) model of TBI (Tehranian et al., 2002).

Polytrauma is common in the context of TBI and leads to profound peripheral inflammatory responses that may exacerbate neuroinflammation. Clinically, patients with polytrauma exhibit increased mortality rates compared to TBI alone: 21.8–11.1%, respectively (Wilson and Tyburski, 2001; McDonald et al., 2016; Sun et al., 2018). Controlled cortical impact (CCI) in combination with tibial injury increases brain levels of IL-1β in mice receiving both injuries compared to those receiving only TBI (Yang et al., 2016). Additionally, FPI in combination with tibial injury corroborates these results, acutely increasing brain levels of IL-1β in mice receiving polytrauma compared to TBI-only controls (Shultz et al., 2015), an effect that may arise from increased peripheral IL-1 expression and transport across the blood–brain barrier (Banks et al., 1993; Plotkin et al., 1996; Sadowska et al., 2015). Polytrauma-induced increases in IL-1β are time dependent however as after 48 h the levels of IL-1β decrease compared to TBI-only controls (Maegele et al., 2007). Sustained inhibition of polytrauma-elicited increases in IL-1 signaling using IL-1Ra reduces cortical neutrophil infiltration, cerebral edema, and brain atrophy following a CHI weight-drop model with concomitant tibial fracture (Sun et al., 2017). It should be noted, however, that IL-1R1 administration did not mitigate behavioral deficits in the MWM, Crawley three-chamber sociability task, open field, or rotarod test (Sun et al., 2017).

The caveat of studies utilizing IL-1Ra lies in the poor ability of IL-1Ra to cross the BBB and the high doses needed to see an effect. Previous preclinical studies have found no changes with low dose, 190 mg/kg IL-1Ra, but did see effects when subjects are administered a high dose of 1900 mg/kg, administered over 7 days (Sanderson et al., 1999). The authors acknowledged that giving nearly 2 g/kg of IL-1Ra is likely to supply large colloid protein load, potentially influencing brain water volumes. Thus, shear amounts of protein could be eliciting an effect independently of IL-1R1 blockade. Pharmacokinetic assessments of IL-1Ra have indicated that brain concentrations are approximately 1% of the plasma concentration at any given time, with a maximum of 2% (Greenhalgh et al., 2010).

If the IL-1Ra therapeutic intervention is pursued, one particular area of importance will be the ability for the molecule to cross the BBB. In fact, some studies have already experimented with modification of the IL-1Ra molecule to enhance its penetration of the BBB. Researchers conducting these studies fused the cell penetrating peptide PEP-1 with IL-1Ra in order to create the novel IL-1Ra–PEP fusion protein. The results of these studies found that the novel fusion protein improved delivery of IL-1Ra due to the molecule’s increased permeability to the BBB compared to unmodified IL-1Ra under the same conditions (Zhang et al., 2017, 2018). Furthermore, modification of the protein did not alter protein function, as evidenced by a significant reduction of inflammatory markers after administration of the fusion protein (Zhang et al., 2017), as well as alleviation of BBB disruption caused by initial injury (Zhang et al., 2018). While these studies focused on treating injuries caused by ischemia–reperfusion, TBIs share many of the negative effects ameliorated by the administration of IL-1Ra–PEP, which suggests that IL-1Ra–PEP would also prove useful for treating the negative effects caused by TBIs. Additionally, since increased permeability of IL-1Ra–PEP across the BBB was shown to occur whether or not the BBB was disrupted, modification of IL-1Ra would address any issues of decreased efficacy in the unmodified therapeutic due to a decrease in permeability after amelioration of the disrupted BBB.

### Genetic KO Models

The role of IL-1R1 has been investigated utilizing genetic knockout models of the receptor. Early experiments involved mice with a global IL-1R1 knockout (Chung et al., 2018). Knockout mice showed increased performance on some behavioral tests in a CHI and a CCI model compared to their WT counterparts. In a CHI model, constitutive, global elimination of IL-1R1 resulted in a normalization of TBI-induced memory deficits in hidden and visible MWM platform trials, resulting in performance comparable to that of the WT sham mice (Chung et al., 2018). In a Y-Maze study, WT CHI mice had a decrease in alternating decisions compared to their respective sham counterparts, whereas IL-1R1 KO mice performed similarly to WT sham subjects (Chung et al., 2018). No overt effects of IL-1R1 elimination alone were found in any of the behavioral paradigms (Chung et al., 2018), providing evidence that inflammatory signaling mediated by IL-1R1 is involved in spatial and working memory deficits following CHI. It should be noted that due to the constitutive nature of IL-1R1 elimination in these subjects the temporal requirements for IL-1R1-mediated signaling in driving these effects are currently unknown.

Subsequent studies have focused on the role of IL-1R1 in various cell types by utilizing a genetic restoration strategy (IL-1R1 Restore mice) (Liu et al., 2015), which is able to selectively restore the expression of IL-1R1 in a cell-specific manner. Global knockout of IL-1R1 and selective restoration to microglia revealed mitigation of IL-1-driven microglial activation following intracerebroventricular (ICV) IL-1β injections (Zhu et al., 2019). Selective deletion of IL-1R1 in microglia had no effect on this process, showing an IL-1-driven microglial activation independent of microglial IL-1R1 (Zhu et al., 2019). In mice expressing IL-1R1 only in endothelial cells, mRNA expression of microglial cytokines, including IL-1β, is increased compared to WT controls (Liu et al., 2019). In a mouse model of cerebral ischemia, pan-neuronal, and brain endothelial-specific IL-1R1 deletion showed reduced infarct volume, with endothelial elimination attenuating BBB permeability and improving neurological function (Wong et al., 2019). Elimination of IL-1R1 in cholinergic neurons also improved functional outcomes and reduced infarct size, providing evidence that specific neuronal populations may be required for IL-1R1-mediated effects within the CNS (Wong et al., 2019). Another notable feature of IL-1-mediated signaling is the ability to drive sickness behaviors. Endothelial IL-1R1 expression has been found to be both necessary and sufficient to induce IL-1-driven sickness behavior in mice (Liu et al., 2019). Combined, these data have begun elucidating the role of IL-1R1 in various cell types of the CNS; however, further experiments are needed utilizing these genetic models in conjunction with models of TBI.

### IL-1β and IL-1α Neutralization

Another strategy commonly employed when targeting immunologic targets is the direct inhibition of cytokine and/or chemokines through the use of antibodies aimed at these respective immunologic signaling components. In the context of neurologic disorders, it is possible that this strategy may exert similar effects as IL-1Ra administration while circumventing the issues surrounding IL-1Ra and minimal BBB permeability. With regard to TBI specifically, preclinical attempts to neutralize the actions of IL-1β using anti-IL-1β antibodies in order to mitigate IL-1R1 binding have been made. One TBI study using the CCI model used continuous ICV-delivered anti-IL-1β via osmotic pump at a rate of 0.375 ng/h to its experimental group; treatment began at 5 min post-injury and continued for up to 14 days thereafter (Clausen et al., 2009). When measured at 1–7 days post-injury, IL-1β neutralization was found to attenuate the CCI-induced cortical and hippocampal microglial activation, as well as cortical infiltration of neutrophils and activated T cells (Clausen et al., 2009). Moreover, lesion volume and hemispheric tissue loss evaluated 18 days post-injury were further reduced by IL-1β neutralization (Clausen et al., 2009). MWM evaluation also demonstrated that IL-1β neutralization reduced the significant deficits in neurological cognitive function induced by CCI, though evaluation by the rotarod and cylinder tests showed no significant reduction in induced neurological motor deficits (Clausen et al., 2009).

While the previous study explores the effects of long-term ICV delivery of anti-IL-1β on the effects of TBI, a follow-up study conducted by the same research group delivered a single 4 μg dose of anti-IL-1β intraperitoneally, first at 30 min post-injury, and again at 7 days post-injury (Clausen et al., 2011). Although measurements at 48 h post-CCI found that anti-IL-1β attenuated the TBI-induced hemispheric edema, it did not improve post-injury memory deficits evaluated by the MWM (Clausen et al., 2011). However, up to 20 days post-injury, anti-IL-1β was found to improve visuospatial learning as evaluated by the MWM, as well as to reduce hemispheric tissue loss and to attenuate TBI-induced microglial activation (Clausen et al., 2011). Taken together, these results suggest a temporal dependence for inhibition of IL-1 signaling to attenuate certain memory-associated deficits. With regards to motor function, this study corroborates the results of the group’s previous study, finding that anti-IL-1β had no significant effect on neurological motor deficits as evaluated by the rotarod and cylinder tests (Clausen et al., 2011).

The particular method used in these studies to explore the effects of IL-1 signaling on TBI-induced deficits, nonetheless, has its limits. First, the use of anti-IL-1β antibody relies on increased permeability of the BBB for the antibody to reach the brain and exert its therapeutic effects. If the body’s initial response to the antibody is attenuation of the disrupted BBB while neuroinflammation persists, then further administration of the antibody will fail to exert the desired effect on brain inflammation. Additionally, the anti-IL-1β antibody targets specifically IL-1β; however, freely available IL-1α can still signal through IL-1R1 and exert proinflammatory effects (Brough and Denes, 2015). This unaccounted signaling could confound the results of studies exploring the relationship among IL-1 proinflammatory signaling, secondary physiological effects, and cognitive/behavioral deficits resulting from TBI.

## Clinical Studies

### IL-1Ra

Due to the promising results in many preclinical studies showing the potential benefits of IL-1Ra treatment post-TBI, clinical trials aimed at determining a potential benefit in heterogeneous clinical populations have been initiated (Helmy et al., 2014, 2016). To date, one clinical trial has been conducted aiming to delineate whether IL-1Ra administration may provide beneficial effects in a clinical neurotrauma cohort. Briefly, 20 patients with severe TBI were recruited within 24 h post-injury and given either IL-1Ra or placebo. Shortly after treatment, the IL-1Ra treatment group showed large increases in IL-1Ra in the brain and in blood plasma compared to the placebo group (Helmy et al., 2016). These results indicate that there is some ability for IL-1Ra to cross the BBB; however, even the infiltration of large doses may be limited.

Initial clinical trials have provided evidence that IL-1Ra treatment results in an increase of pro-inflammatory M1-type macrophages, characterized by increased GM-CSF and IL-1β cytokine levels, whereas levels of IL-4, IL-10, and MDC – the cytokines associated with anti-inflammatory M2-type macrophages – are reduced (Helmy et al., 2016). These results counter previous studies showing decreases in IL-1β levels in treated groups (Sun et al., 2017). The classical view is that M1-differentiated cells induce pro-inflammatory responses and M2 macrophages antagonize pro-inflammatory responses (Martinez and Gordon, 2014); however, this view has recently come under increased scrutiny as microglia and/or macrophages exhibit a wide range of phenotypes that may be induced through a variety of insults.

It is important to note that all data involved with this particular clinical trial are from within the first 48 h of treatment, thus the long-term effects of IL-1Ra treatment post-injury must still be studied. Current trials in stage II are reportedly investigating dose effects, and while initial studies have validated the safety profile of IL-1Ra administration post-injury, a great deal of work remains in order to fully assess IL-1Ra efficacy as a clinical therapeutic for TBI.

## Discussion

Currently, it is clear that targeting components of IL-1 signaling post-injury results in favorable outcomes in preclinical rodent models. Initial clinical studies have also shown currently available therapies to be safe for use in TBI populations, but there is currently a lack of data regarding their efficacy. We posit here that further work in the preclinical and clinical arenas is warranted, as each will provide crucial information required to further the potential use of pharmacotherapies targeting IL-1-mediated signaling. We also believe that recently developed molecular tools aimed at delineating the roles of IL-1 signaling, such as IL-1R1*^*loxP/loxP*^* mice and IL-1R1 Restore mice, will prove useful for determining the specific cell types and timelines critical for targeting IL-1 signaling post-TBI, similar to efforts in preclinical models for ischemia (Liu et al., 2015; Robson et al., 2016; Wong et al., 2019). Although IL-1-targeted therapies have been shown to be safe in clinical populations post-injury, the temporal requirements for targeting IL-1 signaling are important, and the optimization of timing post-injury should be analyzed thoroughly for studies moving forward. Concerted efforts between clinical and preclinical researchers will certainly aid in the further development of IL-1-targeted therapies, and as evidenced by studies discussed above, they may result in the first FDA-approved pharmacotherapies for acute TBI.

## Author Contributions

JT and ER contributed by selecting and summarizing the relevant studies. JT, ER, SC, PG, and MR wrote the original sections of the current manuscript. ER and PG created the figure included in the review article.

## Conflict of Interest

The authors declare that the research was conducted in the absence of any commercial or financial relationships that could be construed as a potential conflict of interest.
